# Transcriptomic profiling highlights cell proliferation in the progression of experimental pulmonary hypertension in rats

**DOI:** 10.1038/s41598-024-64251-w

**Published:** 2024-06-18

**Authors:** Ang Luo, Rongrong Hao, Xia Zhou, Yangfan Jia, Changlei Bao, Lei Yang, Lirong Zhou, Chenxin Gu, Ankit A. Desai, Haiyang Tang, Ai-ai Chu

**Affiliations:** 1 College of Veterinary Medicine, Northwest A and F University, Yangling, 712100 China; 2 State Key Laboratory of Respiratory Disease, National Clinical Research Center for Respiratory Disease, Guangzhou Institute of Respiratory Health, The First Affiliated Hospital of Guangzhou Medical University, Guangzhou, 510120 China; 3 Institute of Zoology, Guangdong Academy of Sciences, Guangzhou, 510260 China; 4 Department of Medicine, Indiana University, Indianapolis, IN 46202 USA; 5 Division of Echocardiography, Department of Cardiology, Gansu Provincial Hospital, Lanzhou, 730000 China

**Keywords:** Pulmonary arterial hypertension, RNA sequencing, Cell proliferation, Systems biology, Biomarkers, Diseases, Molecular medicine

## Abstract

Pulmonary arterial hypertension (PAH) is a progressive disease characterized by pulmonary vascular remolding and occlusion, leading to the elevated pulmonary arterial pressures, right ventricular hypertrophy, and eventual heart failure if left untreated. Understanding the molecular mechanisms underlying the development and progression of pulmonary hypertension (PH) is crucial for devising efficient therapeutic approaches for the disease. Lung homogenates were collected weekly and underwent RNA-sequencing in the monocrotaline (MCT)-induced PH rat model to explore genes associated with PH progression. Statistical analyses revealed 1038, 1244, and 3125 significantly altered genes (P < 0.05, abs (log_2_fold change) > log_2_1.5) between control and MCT-exposed rats during the first, second, and third week, respectively. Pathway enrichment analyses revealed involvement of cell cycle and innate immune system for the upregulated genes, GPCR and VEGF signaling for the downregulated genes. Furthermore, qRT-PCR validated upregulation of representative genes associated with cell cycle including *Cdc25c* (cell division cycle 25C), *Cdc45, Top2a* (topoisomerase IIα), *Ccna2* (cyclin A2) and *Ccnb1* (cyclin B1). Western blot and immunofluorescence analysis confirmed increases in PCNA, Ccna2, Top2a, along with other proliferation markers in the lung tissue of MCT-treated rats. In summary, RNA sequencing data highlights the significance of cell proliferation in progression of rodent PH.

## Introduction

Pulmonary hypertension (PH) encompasses a group of diseases characterized by a mean pulmonary arterial pressure exceeding 20 mmHg at rest in patients^1^. Based on pathophysiological mechanisms, PH is classified into five groups, including Group 1 pulmonary arterial hypertension (PAH), Group 2 PH due to left heart disease, Group 3 hypoxia or lung disease induced PH, Group 4 chronic thromboembolic disease, and Group 5 PH which is a collection of miscellaneous disease-associated conditions^1^. The pathological hallmarks of Group 1, PAH, including arterial intimal thickening, medial hypertrophy, and plexiform lesions^2^, are closely associated with enhanced proliferation and reduced apoptosis of pulmonary arterial smooth muscle, epithelial, and fibroblast cells^3^.

The elevated pulmonary arterial resistance (PVR) in PH leads to right ventricle hypertrophy and can culminate in right heart failure, a major cause of death in PAH patients ^3^. Understanding the intricated molecular mechanism driving PVR and PH progression is essential for advancing diagnostic and therapeutic strategies for the diseases. Prior research has highlighted various signaling pathways and key genes in the development of PH, including the transforming growth factor beta pathway^4,5^, hypoxia-induced factors^6,7^, protein kinase B (AKT)^8^/mammalian target of rapamycin (mTOR) complex^9–11^ and fructose-2,6-bisphosphatase 3^12^. However, given the complexity of PAH, a longitudinal approach beyond cross-sectional or single timepoint investigations can be helpful to elucidate disease progression. High throughput RNA-sequencing (RNA-seq) offers a powerful avenue for genome-wide exploration of genes associated with PH progression^13^. Previous studies identified potential therapeutic targets like ADGRG6 (Adhesion G Protein-Coupled Receptor G6), through the use of RNA-seq^14^ and highlighted upregulated genes such as Kng1(kininogen 1) and Fgg (fibrinogen gamma chain) in lung tissues from PH rats using single time point approaches^15^.

In this study, we performed serial RNA-seq to analyze the transcriptome of the lung tissue from rats treated with monocrotaline (MCT)^16^ at 1, 2 and 3 weeks, identifying 1038, 1244, and 3125 significantly changed genes (P < 0.05, abs(log_2_fold change) > log_2_1.5) respectively at each time point. Pathway enrichment analysis revealed functional associations with pathways such as cell cycle, innate immune system, and G protein coupled receptor (GPCR) signaling. Furthermore, validation via qRT-PCR, Western blot, and immunofluorescence analysis corroborated the increases in cell proliferation markers in lungs from MCT-treated rats. This transcriptomic profiling highlights enhanced cell proliferation during rodent PH progression, providing insights into potential therapeutic targets for PH.

## Results

### Transcriptomic analysis of lung tissues from pulmonary arterial hypertension model rats

**Figure 1:**
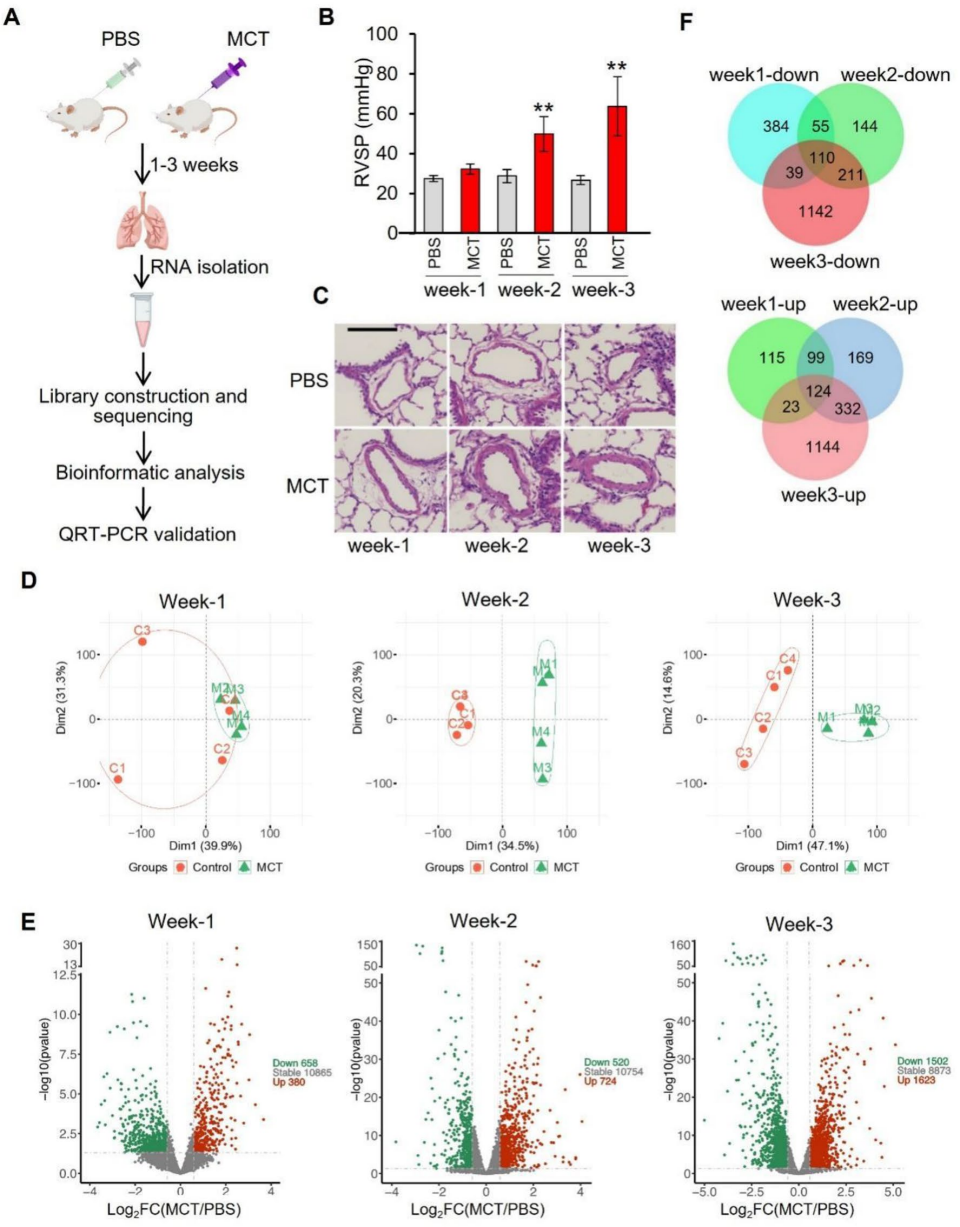
RNA-sequencing analysis revealed differentially expressed genes between MCT rats and control rats. (**A**) Schematic overview of the experimental workflow. (**B**) Right ventricular systolic pressure (RVSP) of the rats were measured by heart catheterization. Results represent mean ± SD, ** P < 0.01, n = 4. (**C**) Representative HE staining images of lung sections from rats treated with MCT or PBS buffer. Scale bar = 100 μm. (**D**) Principal component analysis of RNA-sequencing data. (**E**) Volcano plots showing differentially expressed genes between rats treated with MCT and PBS. (**F**) Venn diagrams showing the number of significantly changed genes shared by rats treated with MCT for different time periods. Week 1-down indicates genes significantly downregulated in the lung tissue of rats treated with MCT for one week.

Rats underwent treatment with MCT or vehicle for one, two- or three-weeks duration. At each time point, the rats were characterized for PH phenotypes including right ventricular systolic pressure (RVSP) lung histology, and RNA/protein isolation (Fig. 1A). The bar graph illustrates that MCT treatment for one week (Week-1) did not significantly affect RVSP. In contrast, MCT treatment for two (Week-2) or three (Week-3) weeks significantly increased RVSP (Fig. 1B). This pattern corresponded with an observable increase in pulmonary arterial wall thickness in rats treated with MCT for two or three weeks compared to control rats (Fig. 1C). These results indicated the successful establishment of the MCT-induced PH rat model.

For RNA-seq analysis, three groups of data were generated in total. Principal component analysis (PCA) for all the detected genes revealed distinct separation between PBS-treated and MCT-treated samples during Week-2 and Week-3 but not for Week-1, suggesting a larger impact on gene expression changes during Week-2 and Week-3 compared to Week-1 (Fig. 1D). This pattern is also reflected by the increasing numbers of differentially expressed genes (DEGs) (P < 0.05, abs (log_2_fold change) > log_2_1.5) from Week-1 (658 downregulated genes and 380 upregulated genes) to Week-3 (1502 downregulated genes and 1623 upregulated genes, (Fig. 1E and Supplementary Table S1). Venn diagrams demonstrated that 110 downregulated genes and 124 upregulated genes were shared between three groups of data (Fig. 1F and Supplementary Table S2), suggesting that the changes of these genes start at the early stage and continue through the later stages during progression of the disease.

### Pathway enrichment analysis of the differentially expressed genes (DEGs)

**Figure 2:**
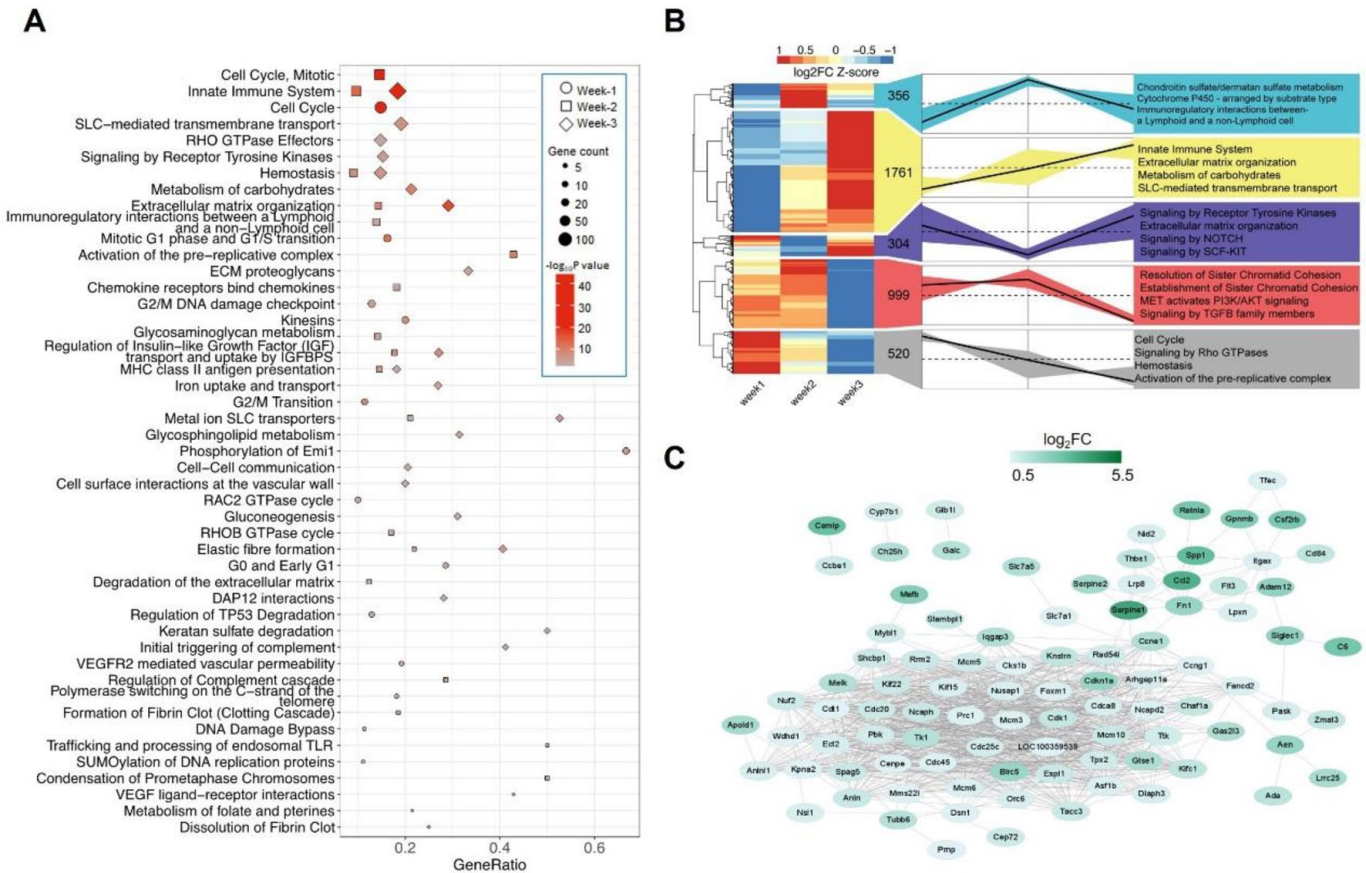
Pathway enrichment and protein–protein interaction analyses for the differentially expressed genes. (**A**) Reactome pathway enrichment analysis of the genes significantly upregulated in MCT rats. (**B**) Hierarchical clustering of fold changes and pathway analysis of significantly changed genes in the lung tissues of MCT model rats. (**C**) Protein–protein interaction network for the common upregulated genes across the three weeks.

Pathway enrichment analysis was respectively applied to these three sets of upregulated genes and downregulated genes by using the Reactome database^17^. Genes upregulated in lung tissues of rats treated with MCT for one week are mainly enriched in cell proliferation-related terms such as cell cycle, mitotic G1 phase and G1/S phase transition, G2/M DNA damage checkpoint, and G2/M transition (Fig. 2A). Genes upregulated in Week-2 and Week-3 are mainly related to pathways like the innate immune system, cell cycle or metabolism of carbohydrase, hemostasis, and extracellular matrix organization (Fig. 2A). The number of pathways enriched by genes downregulated in MCT samples was much smaller. Representative pathways include signaling by GPCR, DAP12 signaling, VEGF signaling, RAC1 GTPase cycle, and signaling by TGFβ family members (Supplementary Figure S1).

In addition, to dynamically analyze the potential function of genes changed during the progression of PH, hierarchical clustering analysis was performed (Fig. 2B). Specifically, genes which continued to change in expression across the three weeks were captured by cluster analysis. Genes which exhibited step-wise increases in expression across each week from weeks 1 through 3 were enriched in pathways like the innate immune system, extracellular matrix organization and metabolism or carbohydrase, while the latter were mainly enriched in the cell cycle, signaling by Rho GTPases and hemostasis.

Base on Venn diagram analysis (Fig. 1F), 110 downregulated genes, and 124 upregulated genes were identified and shared across the three groups of data. To further show the functional association of these common upregulated genes, a protein–protein interaction network was generated for the upregulated genes (Fig. 2C). Most of the proteins corresponding to these common upregulated genes formed close interaction with the other proteins, suggesting that these proteins were functionally relevant. Some of the hub proteins within the network included Iqgap3 (IQ motif containing GTPase activating protein 3), Itgax (Integrin subunit alpha X), Ccne1(cyclin E1), Fancd2 (Fanconi anemia complementation group D2), Tacc3 (transforming acidic coiled-coil containing protein 3) and Anlnl1(actin binding protein-like 1).

### Comparison of the transcriptomic analyses data with previously published proteomic analysis data from MCT-induced PH rat model

**Figure 3:**
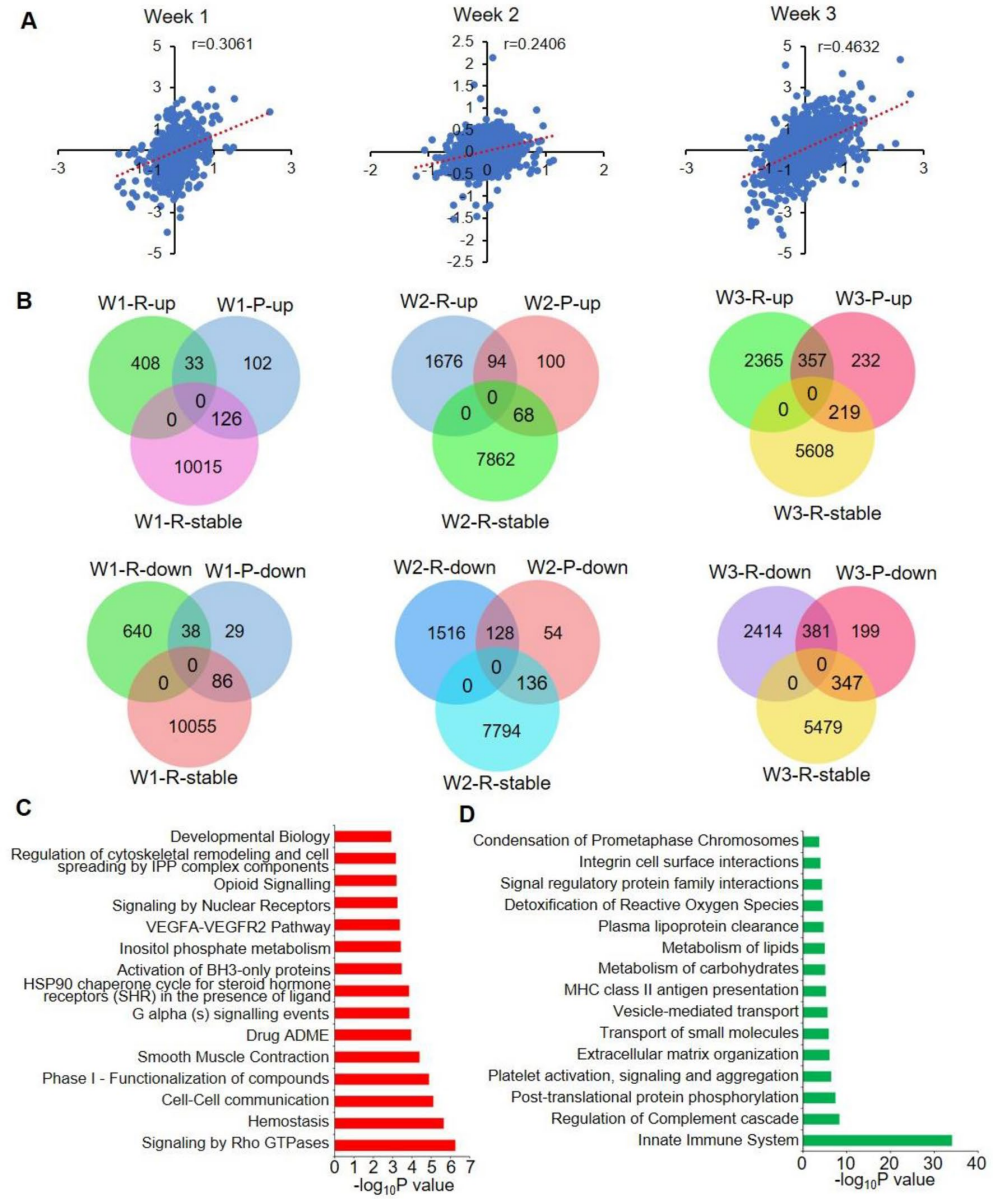
Comparison of the genes identified in this study and the significantly changed proteins identified by published proteomic analysis. (**A**) Correlation analyses of the fold changes of differentially expressed genes and proteins. (**B**) Venn diagrams showing the overlap between differentially expressed proteins and genes. (**C**) Pathway enrichment analyses of the significantly downregulated proteins whose mRNA was also significantly downregulated in MCT rats compared to control samples. (**D**) Pathway enrichment analyses of the significantly upregulated proteins whose mRNA was also significantly upregulated in the MCT rats compared to control samples. Proteins or genes changed in at least one week was included for analysis.

Recent published work characterized a quantitative proteomic analysis of lung tissues from MCT-induced PH rat models and identified several groups of differently expressed proteins between the MCT rats and control rats^18^. We analyzed overlap between the differentially expressed genes in the current study and differently expressed proteins from this prior study. Correlation analyses of the fold changes showed positive but weak correlation between the RNA-seq data and proteomic data, highlighting potiential roles of post-transcriptional regulation in gene expression (Fig. 3A). In fact, for all three groups of data only a small portion of upregulated or downregulated proteins reflected changes in their corresponding mRNAs (Fig. 3B). To study the functions of these gene-protein pairs whose expression changed at both the protein level and mRNA level, pathway enrichment analysis was performed. All of the upregulated and downregulated genes identified in Weeks 1, 2, 3 were respectively pooled together for this analysis. Downregulated gene-protein pairs were associated with pathways such as signaling by Rho GTPases, hemostasis, cell–cell communication and smooth muscle contraction (Fig. 3C), while the upregulated gene-protein pairs were mainly involved in pathways such as innate immune system, post-translational protein phosphorylation, extracellular matrix organization and metabolism of carbohydrates (Fig. 3D).

### Validation of differentially expressed genes

**Figure 4:**
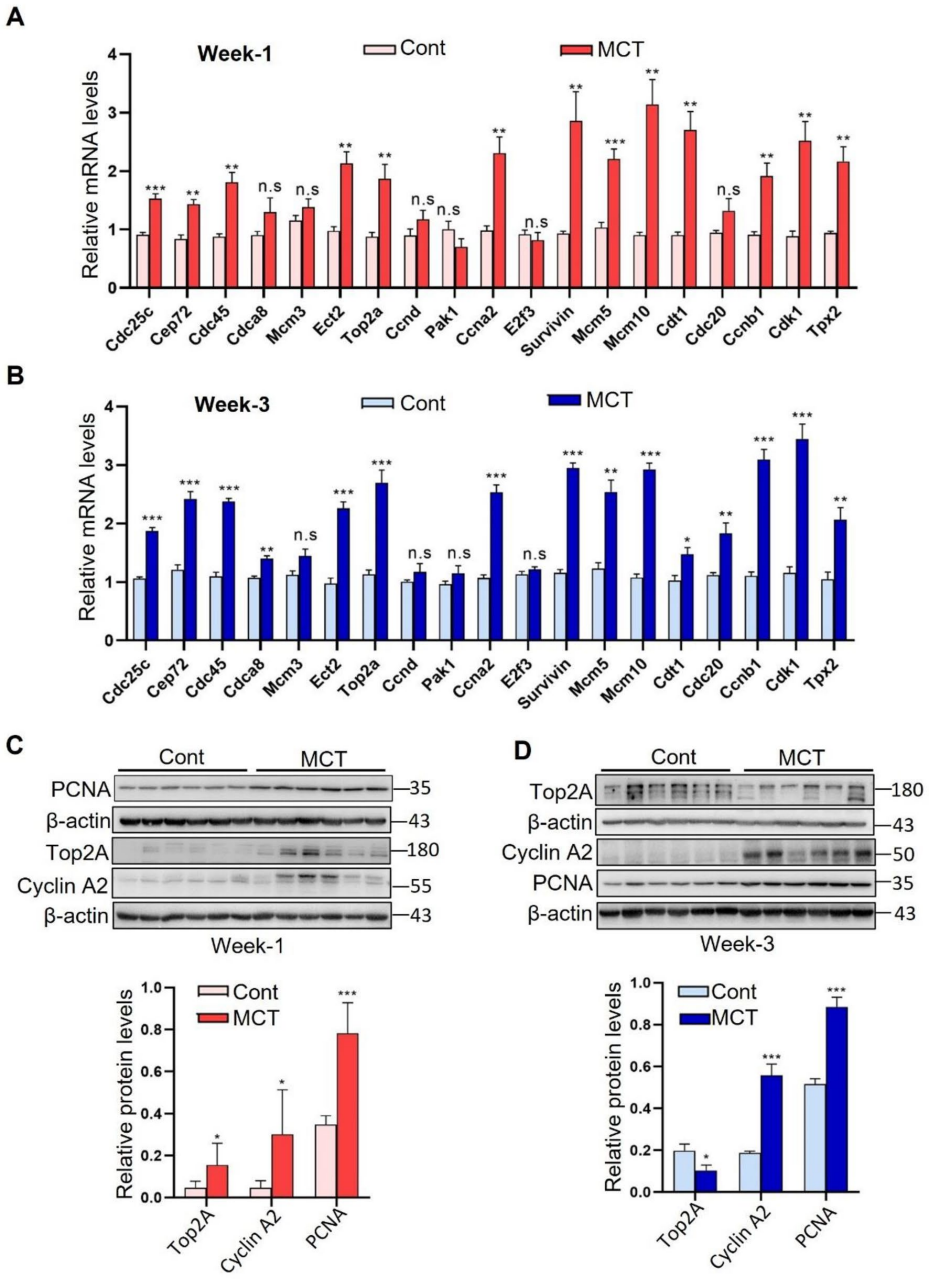
Validation of the significantly upregulated genes identified by RNA-sequencing analysis. (**A**, **B**) Expression of representative significantly upregulated genes identified by RNA-sequencing analysis in the MCT rats were determined by using qRT-PCR at one (**A**) or three weeks (**B**). (**C**, **D**) Western blot analysis and quantification of the indicated cell proliferation-associated proteins at one (**C**) or three weeks (**D**). For A-D, data represent mean ± SD, *P < 0.05, ** P < 0.01, *** P < 0.001, n.s = not significant.

To confirm the DEGs identified by RNA-seq analysis, the expression of representative genes upregulated in all three groups was examined by using qRT-PCR in lung tissues from rats treated with MCT or PBS for one or three weeks. Genes involved in cell cycle transition or proliferation were chosen, such as *Cdc25c* (cell division cycle 25C), *Cdc45* (cell division cycle 45), *Top2a* (Topoisomerase IIα), *Ccna2* (Cyclin A2) and *Ccnb1* (Cyclin B1). qRT-PCR analysis showed that mRNA levels of most of these genes were significantly increased in MCT samples compared to the control samples **(**Fig. 4A,B). In addition, western blotting confirmed the protein levels of Ccna2 and proliferating cell nuclear antigen (PCNA) were increased in lung tissue from rats treated with MCT for one or three weeks (Fig. 4C,D). However, compared to the control samples, the level of Top2a protein was higher in the lung tissues of rats treated with MCT for 1 week but lower in rats treated with MCT at 3 weeks.

### Immunofluorescence staining of cell proliferation markers

**Figure 5:**
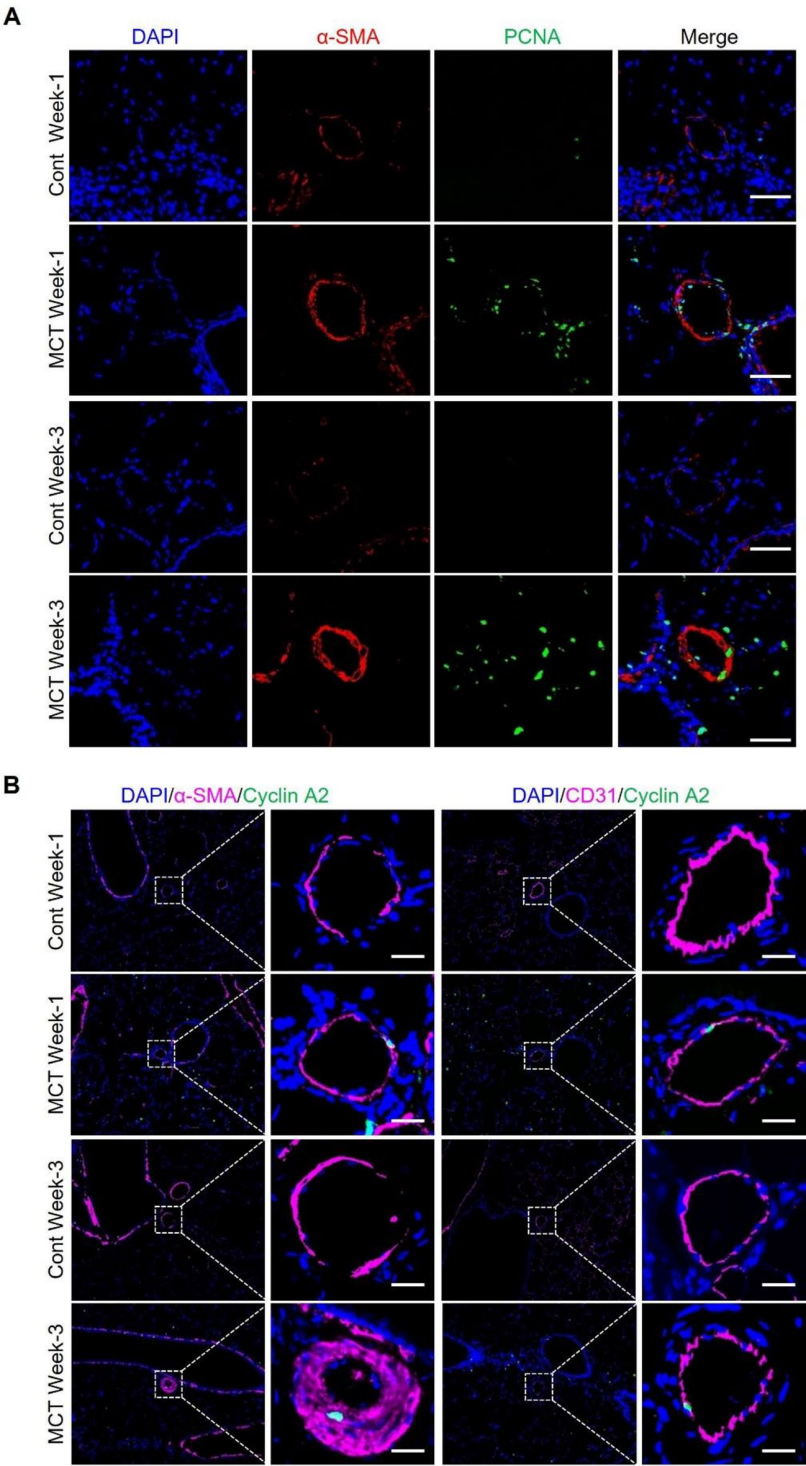
Immunofluorescence staining of cell proliferation markers in the lung tissue of rats treated with MCT or PBS (Cont). (**A**) Immunofluorescence staining of PCNA and α-SMA. Scale bar = 50 μm. (**B**) TSA (tyramide signal amplification)-based immunofluorescence staining of Cyclin A2, α-SMA and CD31. iFluor® 488 tyramide was used for Cyclin A2, Cy5 tyramide was used for α-SMA and CD31. Scale bar = 20 μm.

To test whether cell proliferation was enhanced during the progression of PH, cell proliferation marker proteins including PCNA and Ki67 were stained in the lung tissues of PBS or MCT-treated rats. It showed that the signal of both PCNA and Ki67 was stronger in the MCT rats than that in the control rats, indicating increased cell proliferation in PH (Figs. 5A and Supplementary Figure S2). In addition, immunofluorescence staining also showed increased signal of Cyclin A2 in the lung tissue of MCT rats compared to the control rats (Fig. 5B).

### Clinically relevant genes identified in MCT-rat model

**Figure 6:**
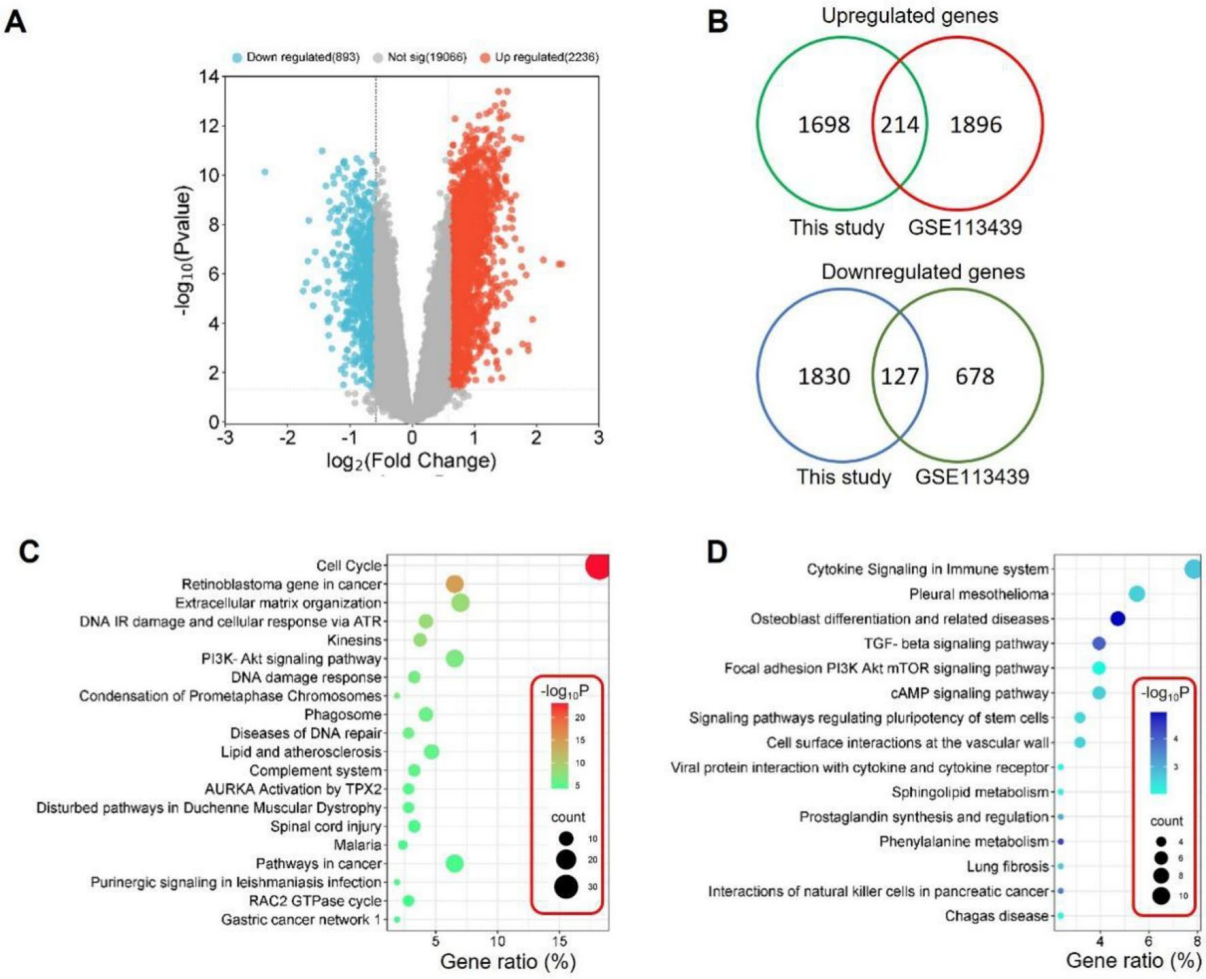
Comparison of the DEGs identified in this study and in the GSE113439 dataset. (**A**) Volcano plot showing the statistical analysis result of the GSE113439 dataset. (**B**) Venn diagram showing the number of common genes between this study and the GSE113439 dataset. (**C**, **D**) Pathway enrichment analysis of the common upregulated genes (**C**) and common downregulated genes (**D**) identified in B.

To test whether the differentially expressed genes (DEGs) identified in the lung tissue of MCT-rats were clinically relevant, the DEGs were compared to the results of a published dataset (GSE113439), which reflected gene expression profiling of lung tissues from 15 PAH patients and 11 normal controls^19^. Statistical analysis of the GSE113439 dataset revealed 893 downregulated genes (log_2_(fold change) < -log_2_1.5, P value < 0.05) and 2236 upregulated genes (log_2_(fold change) > log_2_1.5, P value < 0.05) in the PAH samples compared to the controls (Fig. 6A and Supplementary Table S4). Upregulated genes or downregulated genes in Week-1, 2, 3 were pooled into two groups and respectively compared to the DEGs of GSE113439. Venn diagrams showed that 214 common upregulated genes and 127 common downregulated genes were identified (Fig. 6B and Supplementary Table S5). Pathway enrichment analysis showed that these common upregulated genes were mainly enriched in pathways such as cell cycle, retinoblastoma gene in cancer, extracellular matrix organization, PI3K-Akt signaling pathway and DNA damage response (Fig. 6C), while the common downregulated gene were mainly associated with cytokine signaling in immune system, TGF-beta signaling pathway, and cAMP signaling pathway (Fig. 6D).

## Discussion

The progression of PH involves complex dysregulation of numerous proteins and signaling pathways, posing challenges in developing therapeutic approaches. High-throughput RNA-seq provides an unbiased and powerful method to systematically screen the differentially expressed genes associated with the development of PH and helps to understand the underlying molecular mechanism of this disease. MCT-treated rats and hypoxia-Sugen rats are two of the most popular rat models for PH. These two models share many similarities in mimicking the pathological characteristics of PAH patients, such as pulmonary vascular remolding, increased RVSP, and right ventricular dysfunction^20^. The hypoxia-Sugen model is a powerful translational model, but is limited, in part, by the heterogeneity of its use by investigators including administering different doses of Sugen that are associated with significantly varying timelines (5–11 weeks). Moreover, combined with the low solubility of Sugen and the need of subcutaneous injection, there is a lack of harmonization among laboratories. In contrast, MCT is highly soluble in aqueous buffer, more convenient and largely published as a 3–4 week model. In this study, RNA-seq was employed on the lung tissues from rats treated with MCT or PBS for one to three weeks, aiming to identify genes changed at different stages of PH progression. Statistical analysis revealed 658, 520, and 1502 downregulated genes, and 380, 724, and 1623 upregulated genes at each week, respectively. Among these differentially expressed genes (DEGs), 124 commonly upregulated genes and 110 commonly downregulated genes were identified, many of which have previously demonstrated to be involved in PH, such as Thbs1^21^, CDK1^22^, Foxm1^23^, Ccl2^24^, Nlrp3^25^, and Kpna2^26^, further supporting the reliability of the current RNA-seq analysis. Moreover, in Weeks 2 and 3, a significantly changed gene, Cpa3 (mast cell carboxypeptidase A), recently linked to chronic obstructive pulmonary disease and idiopathic pulmonary fibrosis^27^, emerged as upregulated, suggesting its potential roles in PH.

Pathway enrichment analyses for these DEGs revealed that genes upregulated in Week 1 were highly enriched in cell cycle and proliferation-related pathways. Cell cycle pathway is also a significantly enriched term for genes upregulated in Week 2. Confirmation via qRT-PCR showed increased expression of genes involved in cell cycle progression and proliferation in the lungs of MCT-treated rats. Moreover, western blotting validated upregulation of cell proliferation markers, PCNA and Ccna2, in the lung tissues of MCT-treated rats. Immunofluorescence confirmed the upregulation of PCNA and Ki67, another cell proliferation markers. An intriguing finding was the early-stage upregulation of Top2a, a novel and understudied potential target for PAH. As a major member of the topoisomerase protein family, Top2a play critical roles in DNA replication and cell proliferation, and is upregulated in various cancers^28^, suggesting it’s potential contribution to the development and progression of PH.

The current findings also indicate enhanced cell proliferation is evident at early stage of the disease and progression of PH. Rats treated with MCT for two or three weeks exhibited significantly elevated RVSP than control rats, indicating rapid progression of the disease. Interestingly, these cell cycle markers started to downregulate their expression later in the disease development, which could suggest that once the remodeling is moderate to severe, cell proliferation may not be prioritized in the molecular mechanism of the disease. Genes upregulated during Weeks 2 and 3 were enriched in common PH-related pathways such as innate immune system, hemostasis^29^, and extracellular matrix organization (ECM)^30^. Furthermore, downregulated genes in MCT rats were enriched in pathways associated with PH, including G protein-coupled receptors (GPCRs) signaling^31^, vascular endothelial growth factor (VEGF)^32^ signaling, and collagen degradation, suggesting a potential role of ECM remolding during the progression of PH.

The differentially expressed genes identified in the lung tissues of MCT rats were then compared to the results of a published gene array dataset generated from PAH patients. It showed the overlap between this study and the dataset was between 10%-20%. This observation could partly stem from the difference in species and limitation of the animal model. MCT model for example is associated with toxicity in multiple organs, especially the liver, which is not the case in patients^33^. Therefore, to confirm the association of top genes from the MCT model, our data suggest the need for either additional model evaluation or the need for clinical samples.

In summary, this study comprehensively delineates genes alterations associated with PH progression using RNA-seq in MCT-induced rat models. Pathway enrichment analysis revealed potential pathways involved in diseases progression. Validation through qRT-PCR, western blotting, and immunofluorescence staining strengthens the association of increased expression of cell proliferation-related genes in the lung tissue with PH progression. This work serves as a preliminary exploration, necessitating further in-depth investigations in this field.

## Materials and Methods

All experiments were performed in accordance with the relevant guidelines and regulations approved by the Northwest A&F University and the First Affiliated Hospital of Guangzhou Medical University.

### Animal model

All experimental procedures on animals were approved the by the Animal Care and Use Committee of the Northwest A&F University and the Ethics Committee of the First Affiliated Hospital of Guangzhou Medical University (protocol code 2021070, approval date March 29, 2021). All testing method were reported according to the ARRIVE guidelines^34^. Twenty-four male Sprague Dawley (SD) rats (200 g –250 g) were ordered from Vital River Laboratories (Beijing, China) and housed under regular condition (temperature 20–26℃, humidity 50–70%), all rats have free accessible to water and chow. Two days after reception, the rats were randomly divided into two groups, weighted, and subjected to intraperitoneal injection either with monocrotaline (MCT) (50 mg/kg) or with PBS buffer. During the experiment, the rats were checked every 2 days. One, two, and three weeks after MCT injection, 4 PBS-treated rats (Control group) and 4 MCT-treated rats were randomly chosen and anesthetized one by one with 5% isoflurane for 1–2 min by using an Animal Anesthesia System (RWD Life Science, Shenzhen, China). Then the right ventricular systolic pressure (RVSP) of the rats was measured by using the Millar’s Mikro-Tip catheter transducer (SPR-513). Then the lungs were perfused with PBS to remove the blood in the vessels, and the lung tissues were collected for RNA isolation or histological analysis. The animals were sacrificed by removing the lung and heart under anesthesia with a mask supplied with 2% isoflurane for 5–10 min.

### RNA sequencing

For each rat, about 100 mg of lung tissue was homogenized in 1 ml RNAiso Plus (Takara, Dalian, China). The lung homogenates were centrifuged at 4℃ (14,000 g) for 10 min and the supernatants were further subjected to RNA isolation by phenol–chloroform extraction. mRNA was enriched from total RNA using poly-T oligonucleotide-attached magnetic beads. Sequencing libraries were generated by using the Next UltraTM Directional RNA Library Prep Kit for Illumina (NEB, MA, USA). RNA sequencing was performed by Novogene (Beijing, China) on the Illumina NovaSeq 6000 system.

### Bioinformatics analysis

Raw data were cleaned and then aligned to the rat reference genome (NCBI: GCA_000001895.4) using Hisat2 (v2.0.5). Reads number of each gene was counted the by using the FeatureCounts (v2.0.3). Genes showing an adjusted P value < 0.05 and |log_2_ (fold change) |> log_2_ (1.5) identified by the DESeq2 R package (1.32.0) were assigned as differentially expressed genes. Tidyverse R Package (1.3.1) was used for data processing and visualization. Pathway enrichment analysis was performed online by using the Metascape (https://metascape.org/)^35^. Protein–protein interaction network was constructed based on the STRING database (11.5) and visualized using Cytoscape (3.8.2).

### Quantitative Real-Time PCR (qRT-PCR)

Reverse transcription reaction was performed with 2 mg total RNA in 40 μL volume by using Evo M-MLV RT Kit with gDNA Clean for qPCR II (Accurate Biology, Changsha, China). The reverse transcription product was diluted by ten times, and 2μL diluent was used for each qRT-PCR reaction. QRT-PCR was performed on the CFX Connect Real-Time PCR Detection System (Bio-Rad, Shanghai, China) with the 2 × qPCR SmArt Mix (SYBR Green) (DIYIBIO, Shanghai, China) according to the manufacturer’s instruction. For relative quantification, the expression of each gene was calculated by 2^-∆∆^ method, in which the expression of peptidylprolyl isomerase A (PPIA) was used as the internal control. Primers for qRT-PCR used in the current study are included in Supplementary Table S3.

### Western blot analysis

Total protein from rat lung tissues was extracted in cold RIPA buffer (150 mM NaCl, 50 mM Tris–HCl, pH 7.5, 0.1% sodium dodecyl sulfate, 0.5% NP-40 and 0.5% sodium deoxycholate) containing cocktail protease inhibitor (Thermo Fisher Scientific). Proteins were separated in homemade SDS-PAGE gel and transferred onto PVDF membrane, which was then blocked with 5% non-fat milk (P0216, Beyotime Biotechnology, Shanghai, China) in TBST (Tris-buffered saline + 0.1% Tween 20) for 2 h. Primary antibodies were incubated overnight at 4℃, and HRP-linked secondary antibody (Beyotime Biotechnology, Shanghai, China) was incubated at room temperature for 2–3 h and washed with TBST. The signal was developed with WesternBright ECL HRP substrate (Advansta, Menlo Park, CA, USA) and captured using a chemiluminescent imaging system (Tanon 5200). The following primary antibodies were used in this study: PCNA (Proteintech,10,205–2-AP, Wuhan, China), Cyclin A2 (A7632, Abclone), TOP2A (Proteintech, 24,641–1-AP), actin (Proteintech, 66,009–1-Ig).

### Immunofluorescence

Freshly isolated lung tissues were fixed with 4% paraformaldehyde and then embedded in paraffin. The lung tissue Sects. (5 μm) were prepared for hematoxylin and eosin (H&E) staining or immunofluorescence (IF) with the relevant antibodies. For IF, the lung sections were first deparaffinized in xylene and rehydrated in graded ethanol. Antigen retrieval was performed in sodium citrate (pH 6.0). After blocking with 5% BSA, the sections were incubated with primary antibodies targeting α-SMA (Cy3 conjugate) (Sigma, C6198), PCNA (Proteintech, 10,205–2-AP), Cyclin A2 (Abcam, ab181591), CD31(Abcam, ab182981) at 4 °C overnight in a humidified chamber. The next day the sections were washed with PBST and then incubated with CoraLite488-conjugated Goat Anti-Rabbit IgG (H + L) (Proteintech, SA00013-2) for 1 h in the dark. Finally, all sections are counterstained with an antifade mounting medium containing DAPI (Beyotime, P0131). For co-staining of α-SMA and Cyclin A2, and co-staining of CD31 and Cyclin A2, Tyramide signal amplification (TSA) method was used^36^. iFluor® 488 tyramide (ATT Bioquest, 11,060) was used for Cyclin A2, while Cy5 tyramide (ATT Bioquest, 11,066) was used for α-SMA and CD31. Pictures were captured by using a fluorescence microscope (Leica DM6 B).

### Statistical analyses

Statistical analyses were performed with R Studio, GraphPad Prism or Excel. Differences between treatments were calculated with Student’s t test. Data were presented as mean ± standard deviation (SD), *P < 0.05, ** P < 0.01, *** P < 0.001.

### Supplementary Information

Supplementary Table S1.

Supplementary Table S2.

Supplementary Table S4.

Supplementary Table S5.

Supplementary Information.

## Data Availability

The RNA-sequencing raw data for the current study have been deposited at Gene Expression Omnibus (GEO) with the accession number GSE GSE229361 (https://www.ncbi.nlm.nih.gov/geo/query/acc.cgi?acc=GSE229361).
