# Supplementary material for: Transcriptomic profiling highlights cell proliferation in the progression of experimental pulmonary hypertension in rats
Source: Sci Rep. 2024 Jun 18;14:14056. doi: 10.1038/s41598-024-64251-w (PMC11189536; doi:10.1038/s41598-024-64251-w)
Supplement: Supplementary file 5 — Supplementary Information. [file 41598_2024_64251_MOESM5_ESM.pdf]

Supplementary Information:

Supplementary Figure S1: Reactome pathway enrichment analysis of the genes significantly downregulated in the lung tissue of MCT rats.

Supplementary Figure S2: Immunofluorescence staining of a-SMA and Ki67 in the lung tissue of rats treated with MCT or PBS (Cont). Scale bar=100 mm.

Supplementary Table S1: List of differentially expressed genes

Supplementary Table S2: Venn diagram analysis for the differentially expressed genes

Supplementary Table S3: Primers for qRT-PCR

Supplementary Table S4: Statistical analysis of GEO113439 dataset

Supplementary Table S5: Common genes significantly changed in MCT rats and PAH patients

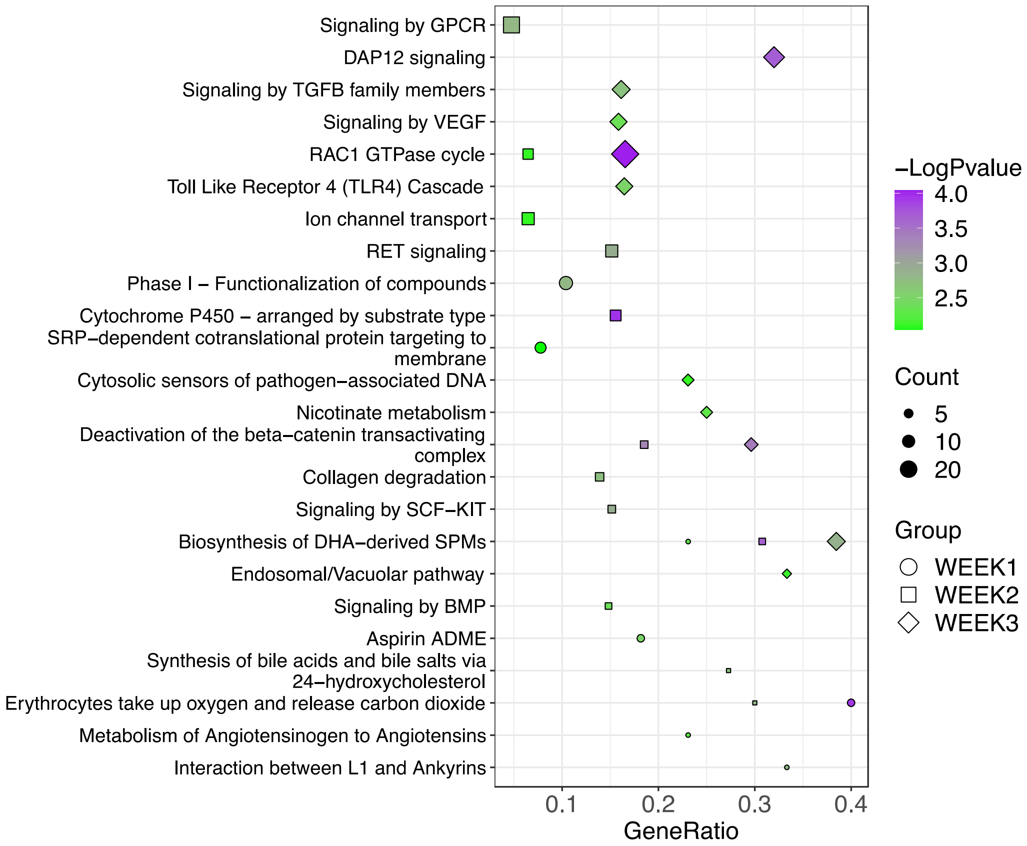

Figure S1. Reactome pathway enrichment analysis of the genes significantly downregulated in the lung tissue of MCT rats.

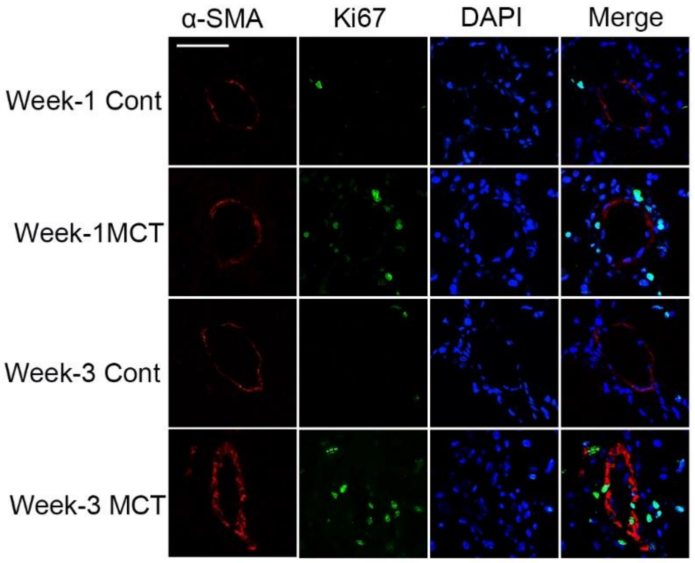

Figure S2. Immunofluorescence staining of α-SMA and Ki67 in the lung tissue of rats treated with MCT or PBS (Cont). Scale bar=100 µm.

Supplementary Table S3

	Primers for qRT-PCR	
**Gene name**	**Forward**	**Reverse**
Cdk1	GGGAACAGAGAGGGTCCGTT	ATTTCCCGGATTGCCGTACT
Cdc20	GAATGCCCCAGAAGGCTACC	TCATTCCGGATTTCAGGGGC
Cdt1	ACGTCCCCACTTTCAAGGAC	GACGCCAGGAAAACCTTGTG
Cdc45	GTCACTGCTGTCCTGAGGAATTT	AATGTTATCTTACAAGTGAACAGCG
Mcm10	CCAACCGTGTCTTAGCCCAG	CATTCTCCTCCGAGCCACAG
Mcm3	CTTGGACTTCCTGGACGACG	TTGTTCAGGAGGCGGTTAGC
Mcm5	GCGGCTCTGTGAGGTACAGT	CGCTTCTGTAGGTGCGACTT
Cdc25c	GCAATGGAGAGGTGTCTGCT	AGAGAAGCTGTGCTGGGATG
Cep72	GAAAATATGGCGCCAGGTCAG	AAGATCGAAGCTCAGCCAGG
Cdca8	CTTTCCCGGTCTCATTCGCT	TGGTTCGAACTTGCACCTCG
Survivin	AGCCAAGAACAAAATTGCAAAGG	CCTGGAAAGCTGGGACAAGT
Ccdn	TCAAGTGTGACCCGGACTG	GACCAGCTTCTTCCTCCACTT
Pak1	AGCGAGCGCAGAAGTAGC	TGGAGGCAGAGGTTTGGAAC
Ccna2	TGGATGGTAGTTTTGAATCACCC	GGCCCGCATACTGTTAGTGA
Ccnb1	ACAACGGTGAATGGACACCA	GCCACGGTTCACCATGACTA
Top2a	CAGCGTGTTGAGCCTGAATG	ATAACTTGGGAGCATGGGCA
E2f3	CGAGAGTGGCCATCAGTACC	TGAGGGAGATTTTGGAGTTTTTGG
Tpx2	GTCAGCTACTAGACGAGGACG	TTGATAAAGTCGGTGGGGGC
Ect2	TGTGGTCAAGCAAGAGTGGTT	CGTCTTTTGCGGTTGCTGTT
Ppia	AGGATTCATGTGCCAGGGTG	CTCAGTCTTGGCAGTGCAGA

Raw Western blot images for Figure 4C

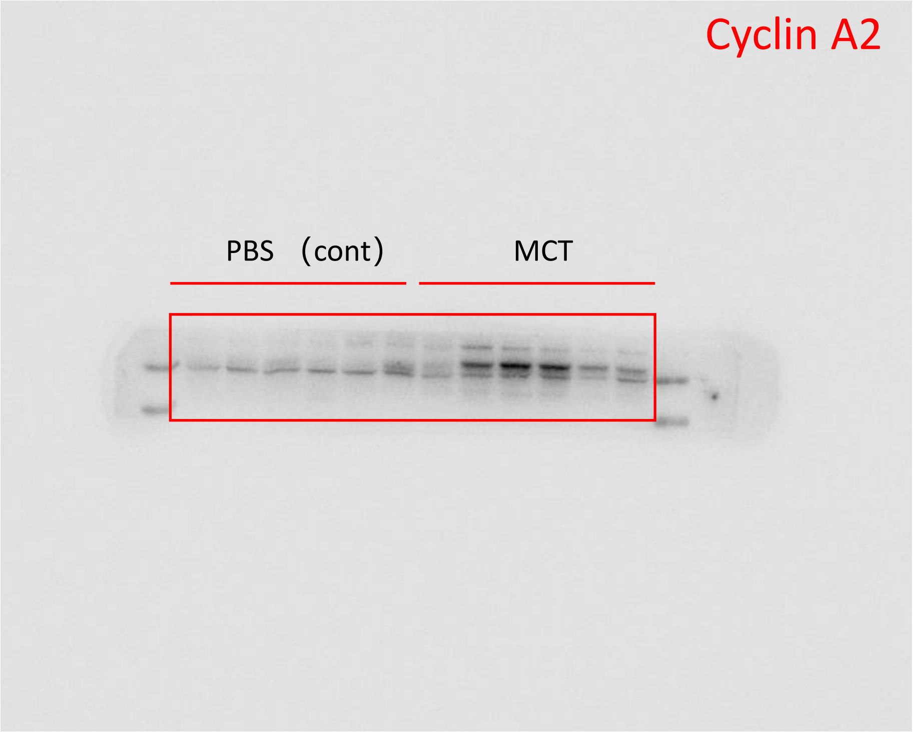

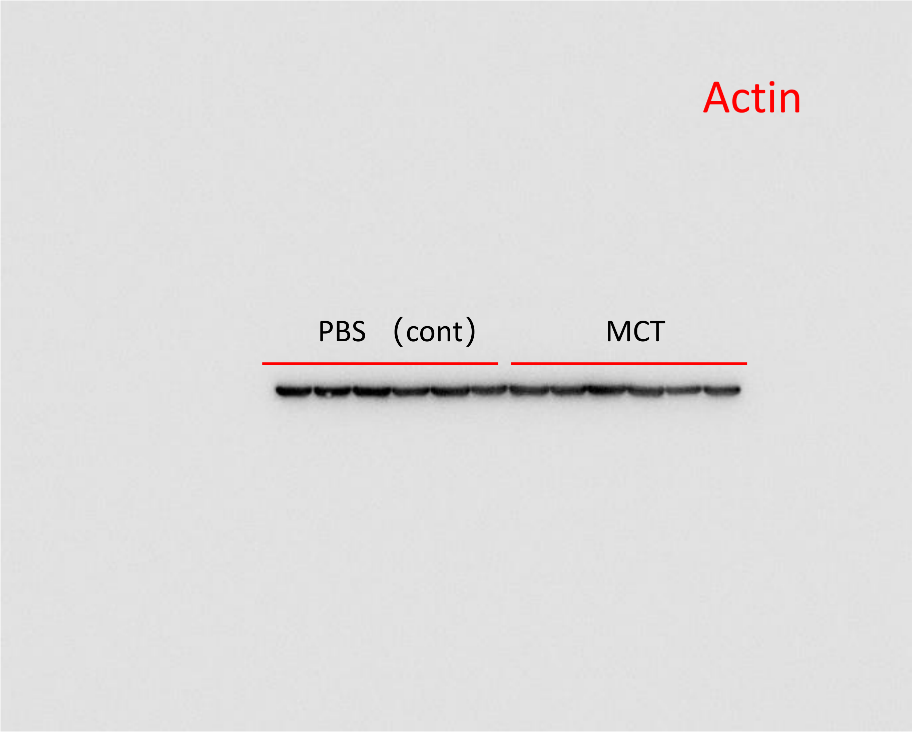

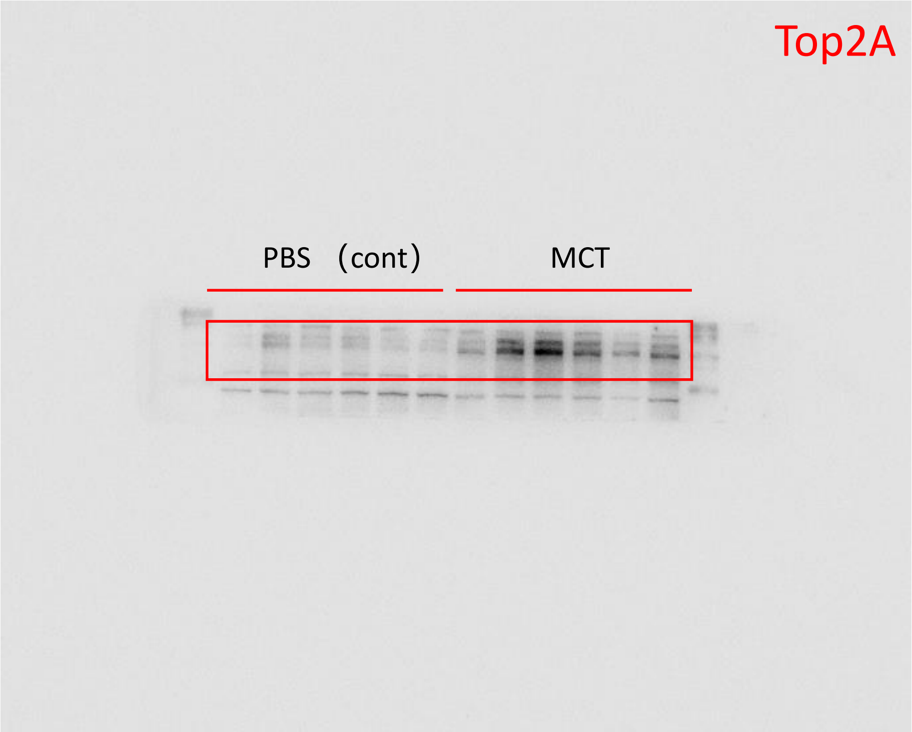
Top2A
PBS (cont)
MCT

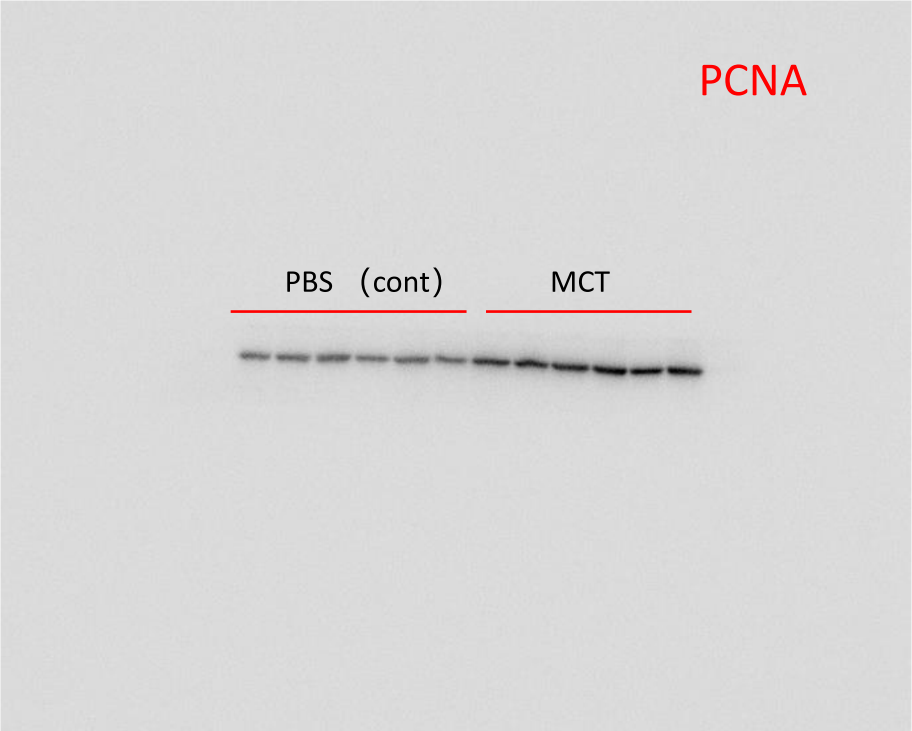
PCNA
PBS (cont)
MCT

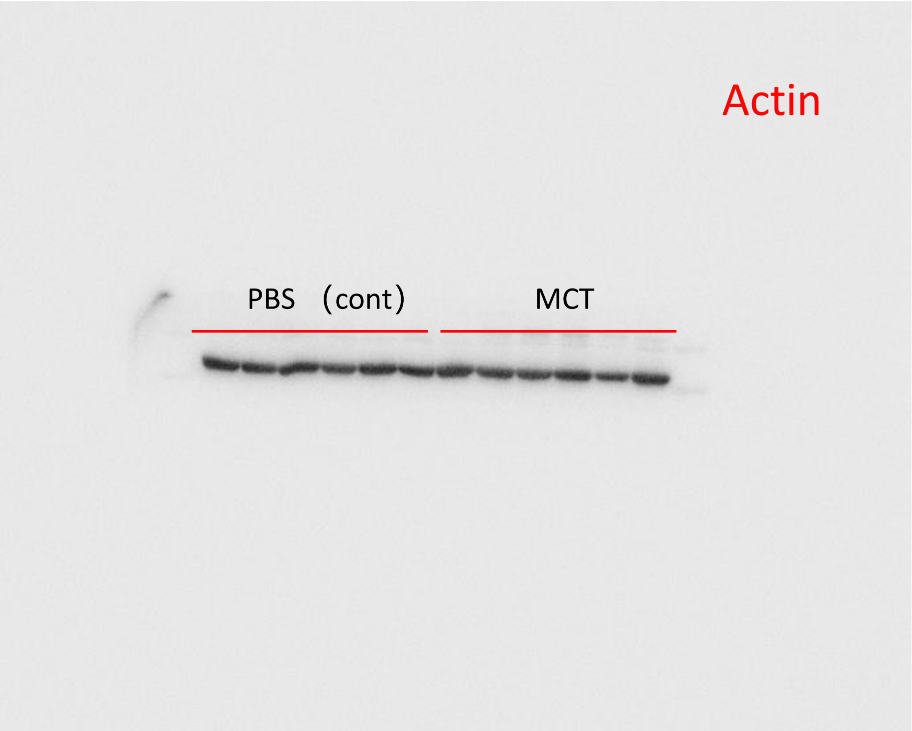
Actin
PBS （cont）
MCT

Raw Western blot images for Figure 4D

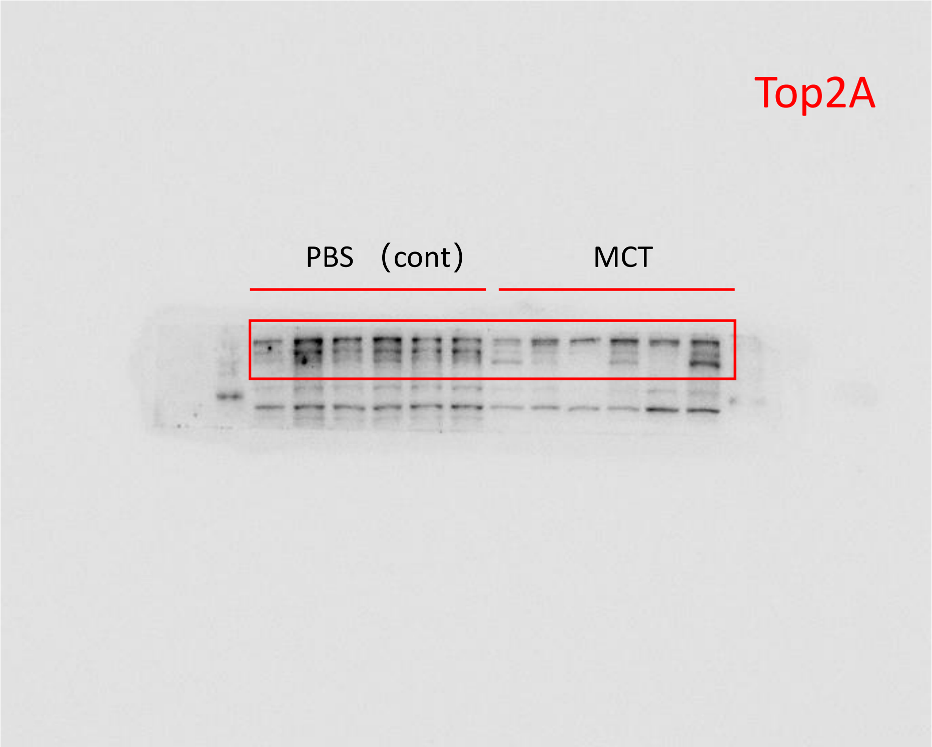

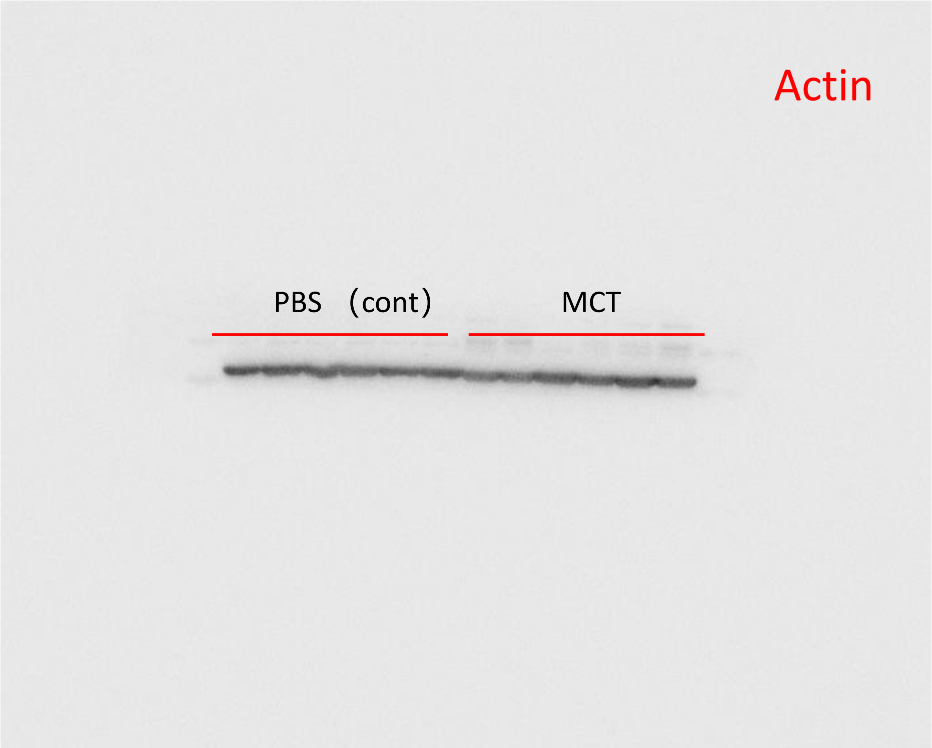

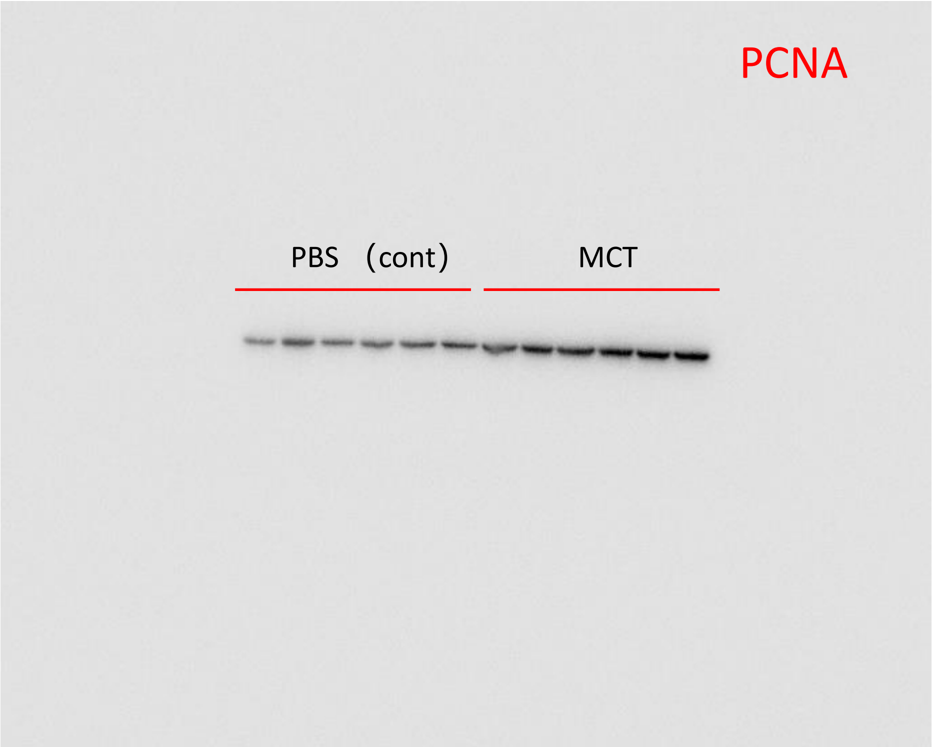
PCNA
PBS (cont)
MCT

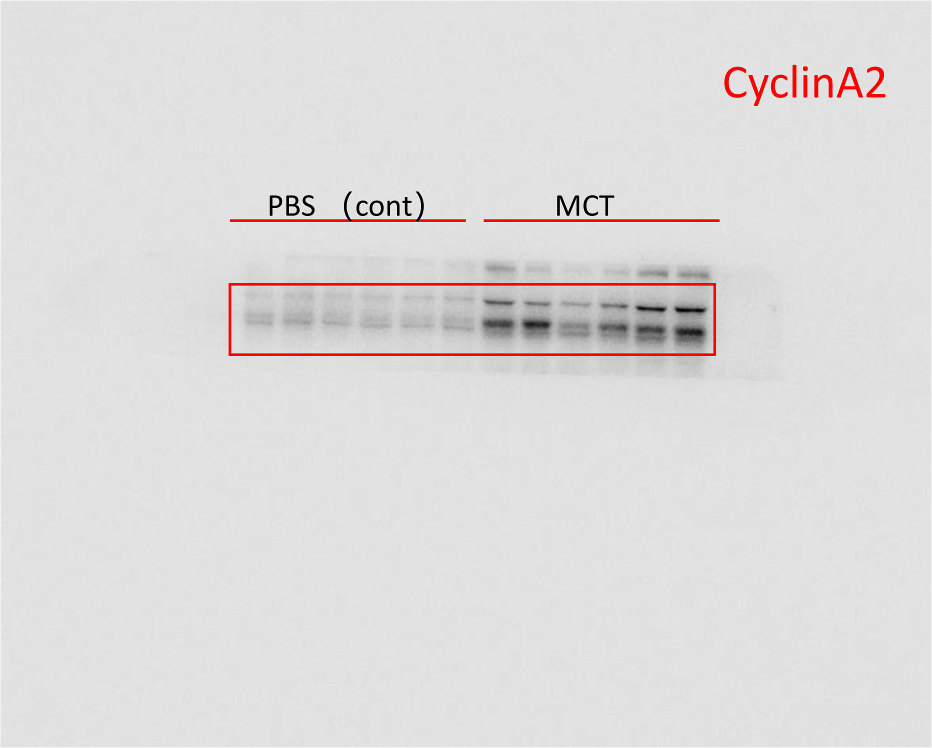
CyclinA2
PBS (cont)
MCT

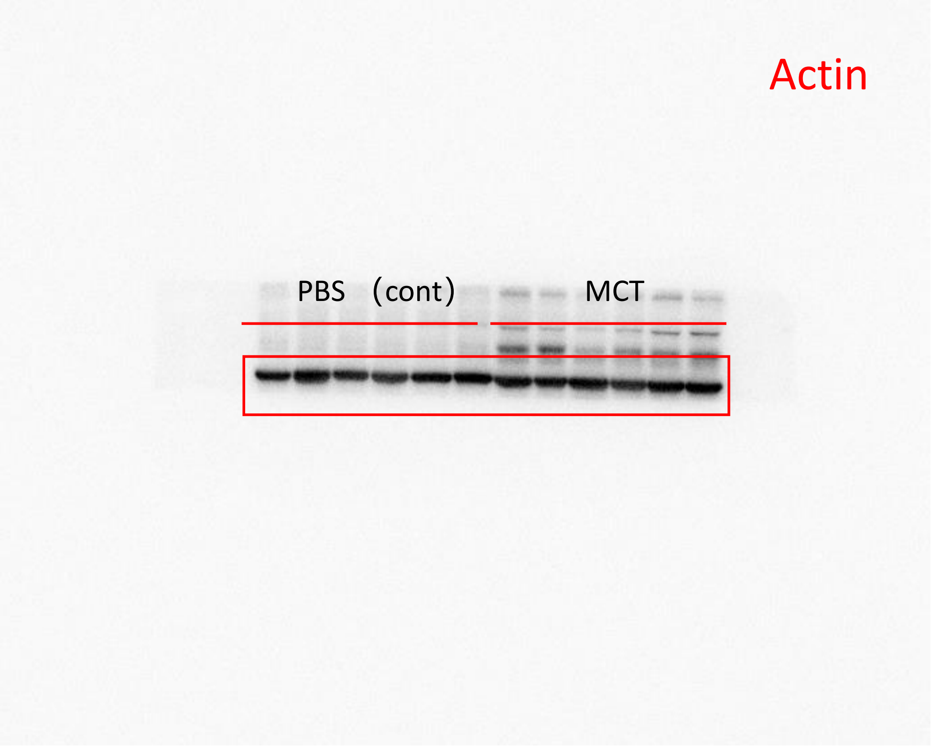
Actin
PBS (cont)
MCT
